# Supplementary material for: Four-Dimensional Stimuli-Responsive Hydrogels Micro-Structured via Femtosecond Laser Additive Manufacturing
Source: Micromachines (Basel). 2021 Dec 27;13(1):32. doi: 10.3390/mi13010032 (PMC8779986; doi:10.3390/mi13010032)
Supplement: Supplementary file 1 [file micromachines-13-00032-s001.zip › micromachines-1487489-supplementary.pdf]

Supplementary materials

Four-dimensional stimuli-responsive hydrogels micro-structured *via* femtosecond laser additive manufacturing

Authors
Yufeng Tao [1,2,*], Chengchangfeng Lu [3], Chunsan Deng [2], Jing Long [2], YunPeng Ren [1], Zijie Dai [1,*], Zhaopeng Tong [1], Xuejiao Wang [1], Shuai Meng [1], Wenguang Zhang [2], Yinuo Xu [2], Linlin Zhou [2]

Affiliations
1 Institute of Micro-nano Optoelectronics and Terahertz Technology, Jiangsu University, Zhenjiang 212013, China

2 Wuhan National Laboratory for Optoelectronics, Huazhong University of Science and Technology, Wuhan 430074, China

3 Whiting School of Engineering, Johns Hopkins University, Baltimore, MD 21218, USA

Corresponding author's email address
Taoyufeng@ujs.edu.cn, 2017508020@hust.edu.cn

Keywords
Femtosecond laser; Additive manufacturing; Hyaluronic acid methacryloyl; Polyethylene glycol diacrylate; Stimuli-responsiveness.

Table of Contents

Abstract

Figure S1.The fluorescence, phase, and merged images of our fabricated hydrogels loading with cells.

Figure S2. The summarized cell viability of as-prepared hydrogels in Figure S1.

Figure S3. Molecular structure of 2-hydroxy-2-methylpropiophenone used as photon initiator.

Figure S4. Dimension measurement on the responsive hydrogels before and after saturated swelling using the Nanomeasurer software 1.2.

Figure S5. The optical intensity distribution of laser voxel at varied focusing positions.

Figure S6. Height measurement using advanced laser confocal microscopy.

Figure S7. Mechanics test platform used for determining the mechanical properties of our hydrogels.

Figure S8. Compressive and tensile test of micro-probe penetrating or pulling out of as-prepared square hydrogel.

Explanation on surface tension theory

Supplementary videos

Abstract

We combine a frequency-doubled femtosecond laser system with writable responsive hydrogel materials for micro/nanoscale self-adaptive machines and actuators. The high-efficiency tool for smart shape-reconfigurable devices is of interest to massively-apply microscopic bio-engineering, actuators, robotics, and micro/nanoelectromechanical systems (M/NEMS). Herein, we successfully prepared a smart photoresist to give the stationery products the dramatic shape morphing ability. Flexible laser parameters, line-to-line writing strategy, ultra-fast photo-chemistry theoretical rationale, material mechanics analysis, and characterization are presented here as guidance for using this material system and fabrication method.

Due to the interpenetrative hydrogel matrix with pre-designed structures, responsive behavior becomes controllable to create specific structures, 2D-to-3D transform, or recovery into initial shape like a shape-memory material. The dynamic gel mechanics also provide a desirable self-standing ability for multi-layer structure.

Compared to conventional mechanical, thermal/electronic- chemistry, soft lithography or self-assembly methods, this new method promises a much-improved structural diversity, spatial resolution, stimuli-responsiveness, freedom of model design, and strong possibility in functional integration. We believe this work extends the conception of light-matter interaction and is easy-duplicated, productive, adjustable in the material system or shape morphing.

Supplementary figures

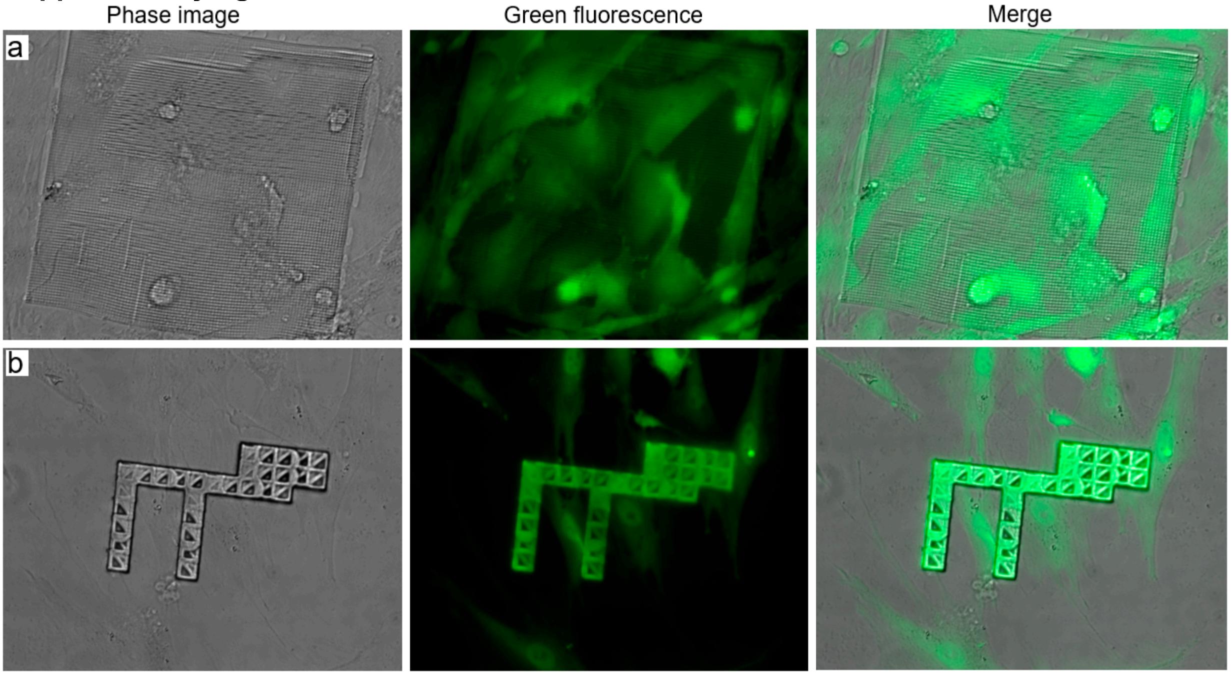

Figure S1. The fluorescence, phase, and merged images of our fabricated hydrogels loading with cells.

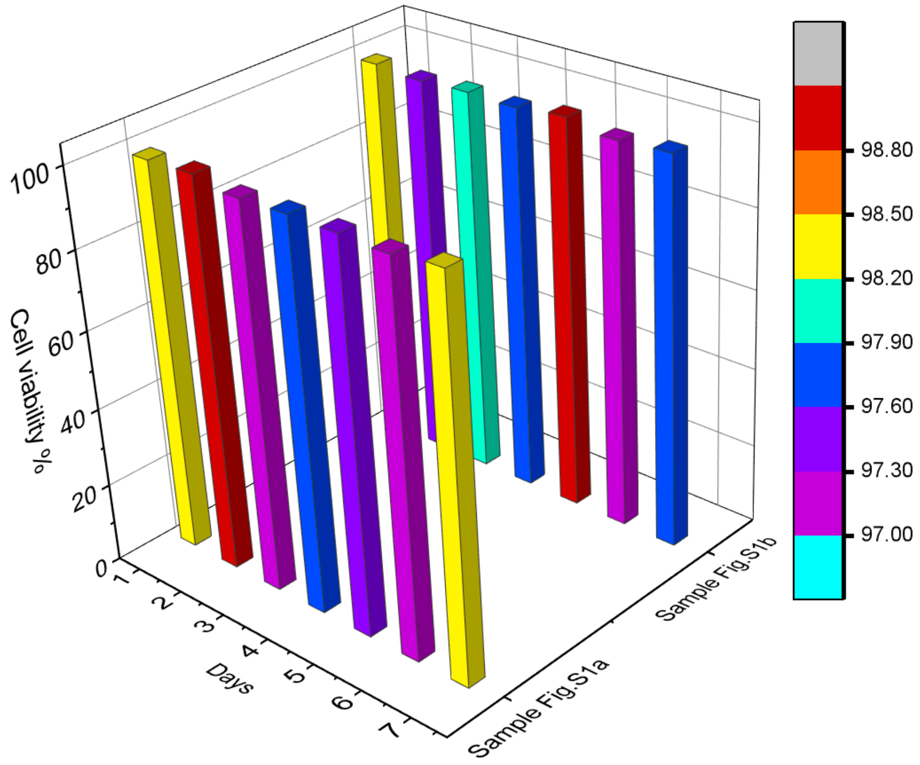

Figure S2. The summarized cell viability of as-prepared hydrogels in Figure S1.

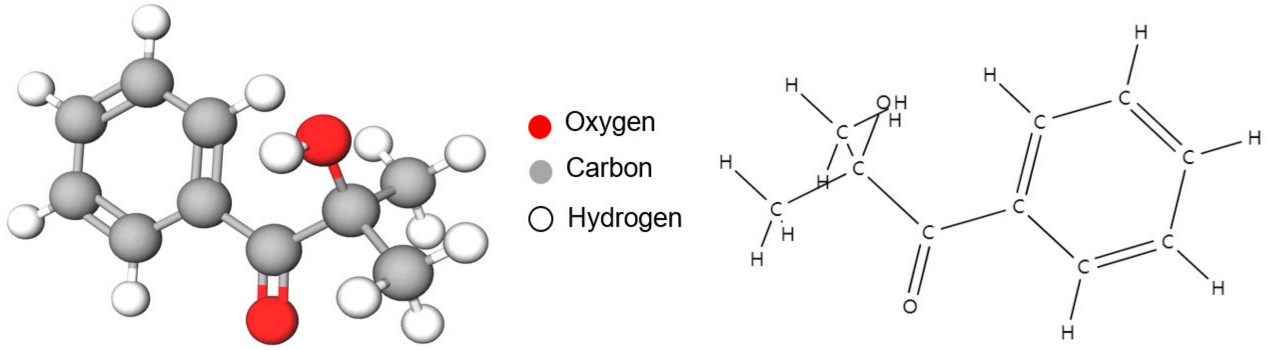

Figure S3. Molecular structure of 2-hydroxy-2-methylpropiophenone used as photon initiator.

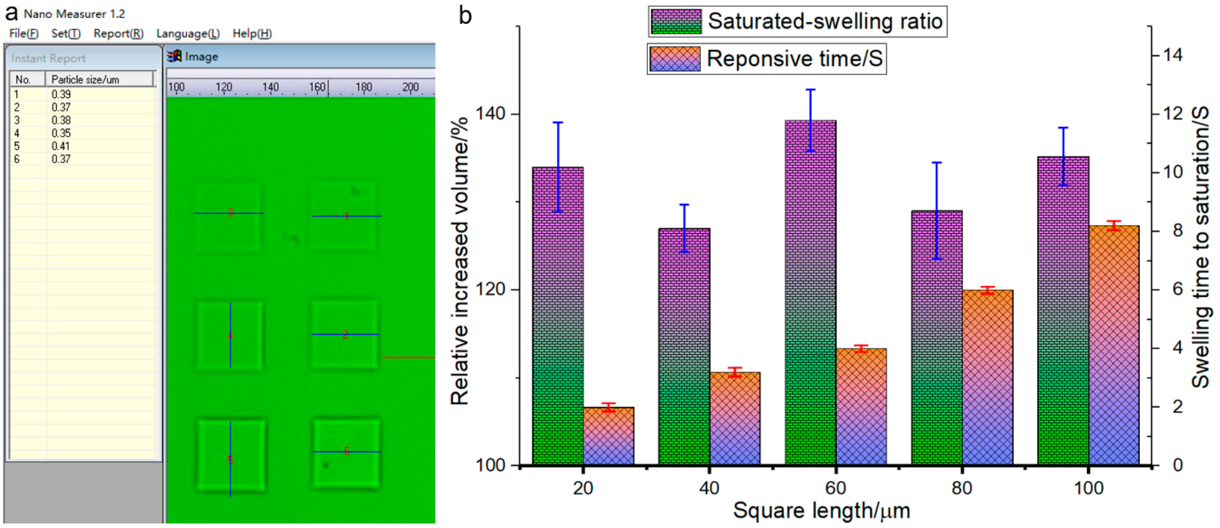

Figure S4. Dimension measurement on the responsive hydrogels before and after saturated swelling using the Nanomeasurer software 1.2. (**a**) The software vision on square-shaped hydrogel. (**b**) By changing the size of hydrogel, we record the relationship of responsive time versus swelling ratio.

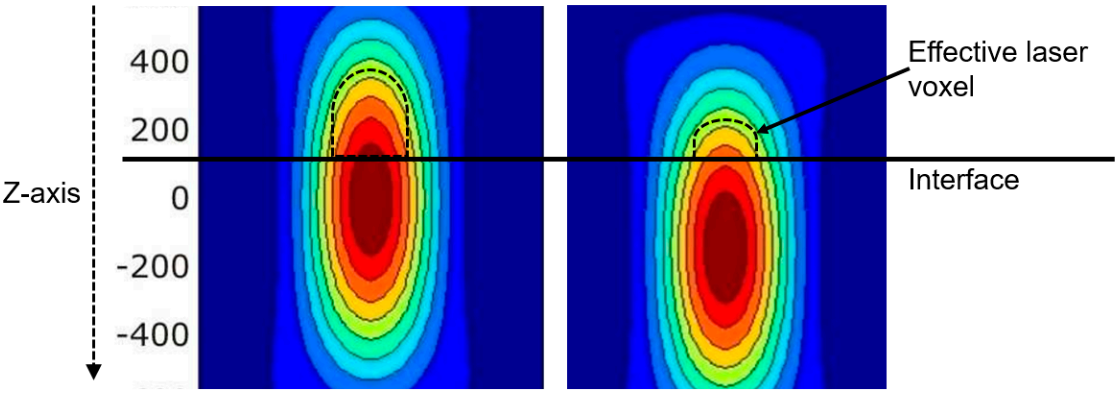

Figure S5. The optical intensity distribution of laser voxel at varied focusing positions.

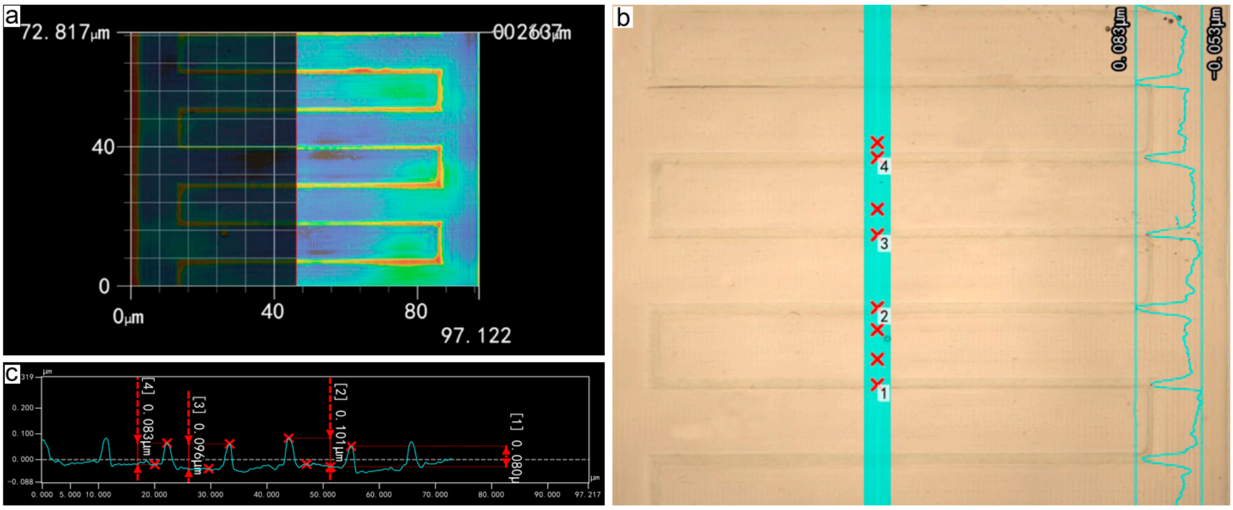

Figure S6. Height measurement using advanced laser confocal microscopy on the fabricated hydrogels.

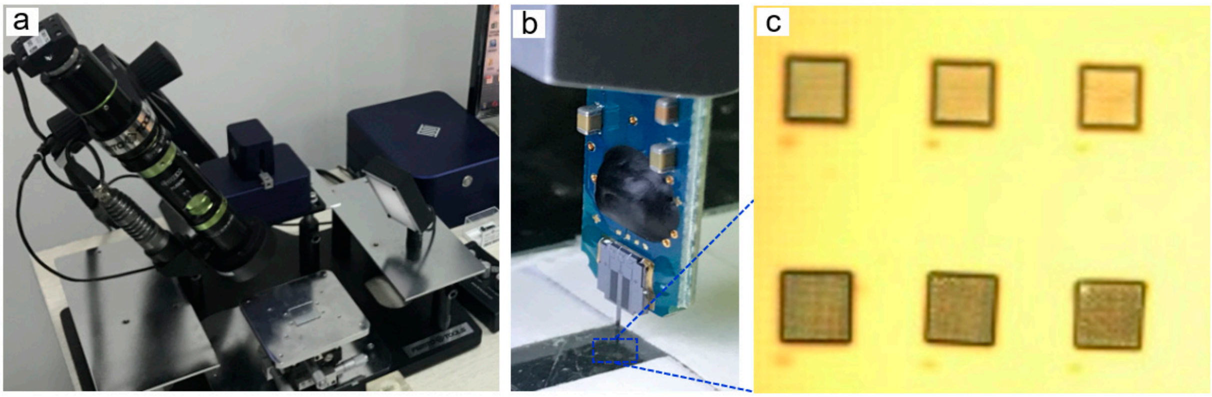

Figure S7. Mechanics test platform used for determining the mechanical properties of the square hydrogels. (**a**) The sample was vertically penetrated or pulled out using an FT-S1000 microprobe (50×50 μm tip radius) with a 50 nN resolution and an electronic capacity force sensor; (**b**) the micromechanics testing system consisted of a microscope, control box, and maneuvering robotics with 0.2 nm precision; (**c**) the tested square-shaped hydrogel.

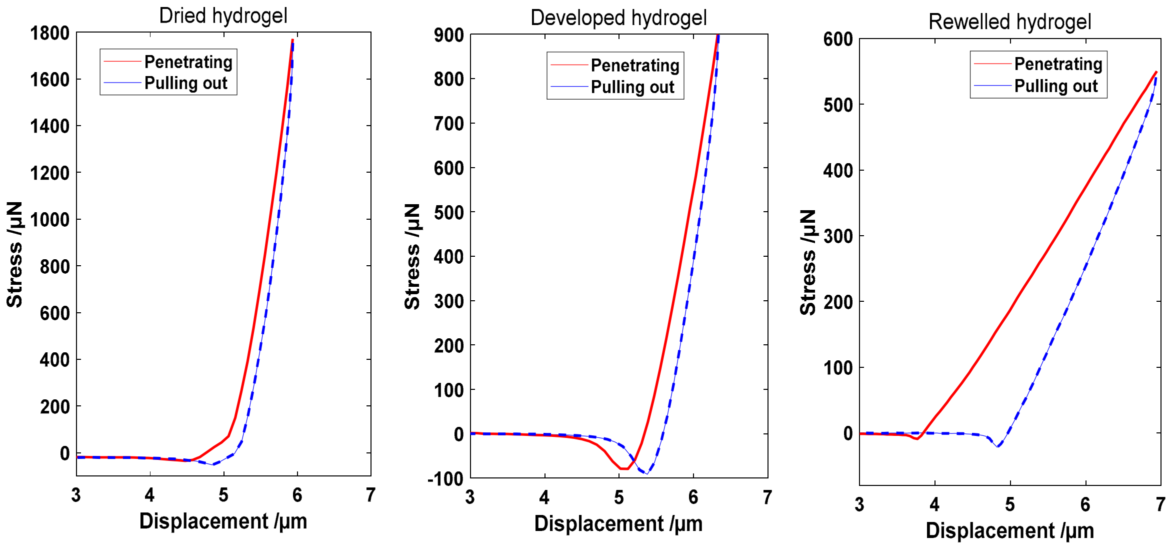

Figure S8. Compressive and tensile test of micro-probe penetrating or pulling out of as-prepared square hydrogel. Hysteresis loop composed of stress-to-displacement data in a round-trip movement of the microprobe; The calculation of E is accomplished by averaging the slopes of the red and blue curves. The left figure is on the dried hydrogel with a higher E. The middle figure is the stress-to-displacement points on the hydrogel just-developed. The right figure is on the equilibrium-swelled sample with a lower slope.

Explanation on surface tension theory

In as-demonstrated shape morphing, the mesoscopic variation of Young's modulus is accumulated to generate macroscopic tensile force for the reshaping process. In consecutively scanned nanowires, linewidth ΔL of the laser scanned nanowire (NW) is often 0.5~2.5 times of nanowire width (ΔN). Young's modulus of spacing is altered much more than the array of NWs to generate a bending moment, ΔM, and release residual stress, Δσ. A force model associated with E is proposed to expound the surface tension generated in shape reconfiguration. In Equation (S1) (I= moment of inertia, unit: Kg•m^2; ρ = radius of curvature in the beam deflection theory, unit: μm-1), and declining -ΔE created a negative bending moment ΔM (unit: N•m) over the rectangle sheet. Surface tensile force ΔF is an integration of residual stress, Δσ, (unit: N) on single-layer thickness H along with the entire length L (Equations (S1) and (S2), ε = local strain, v = Poisson ratio of the fabricated gelation).

$$\Delta M = -\Delta E I/\rho \qquad \text{(S1)}$$

$$\Delta\sigma = -\Delta E\varepsilon/(1-v) \qquad \text{(S2)}$$

$$\Delta F = \int_0^H L\Delta\sigma \mathrm{d}z \qquad \text{(S3)}$$

As revealed in Equations (S1)-(S3), the surface tension, ΔF, induced by the swelling-produced expansion was outward and perpendicular to the NWs. However, shrinkage-produced surface tension, ΔF, is inward and perpendicular to the NWs, and the positive ΔM is more likely to bend at two edges to form a "C" shape. If ΔF is weaker than adhesive force, hydrogels underwent in-plane swelling or shrinkage. Otherwise, out-plane bending occurred. Bending

angles of swelling and shrinkage both follow an exponential time relationship at a constant temperature (Equations (S4) and (S5)). The time-dependent bending angle is denoted by β(t), t denotes time, τ is a bending time constant determined by material and temperature, and L denotes the lateral bending length. The curvature, κ, observed can reach 1 μm^{-1}.

$$\beta(\mathrm{t}) = \beta_0\left[1 - \exp\left(\frac{t}{\tau}\right)\right] \qquad \text{(S4)}$$

$$\kappa = \beta/L \qquad \text{(S5)}$$

Young's modulus affects the bending direction and degree of responsiveness. Therefore, we use an ultrafine, water-proof, force-sensing probe (FT-MTA2, FemtoTools, Seen in supplementary materials, Figure S7) at 0.01 μm minimum step to penetrate into and pull out of hydrogel to conduct compressive and tensile tests to determine Young's modulus. In 20 cycles of the test, synchronous stress, ΔS (load force, unit: μN), and probe tip displacement, ΔD (Unit: μm), were collected. Assuming a Poisson's ratio of v = 0.49 for the hydrogel, Young's modulus, E, was approximately (1.150 ± 0.22) × 10^3 MPa after the alcohol development, decreased to (0.42~0.75) × 10^3 MPa when immersed in water, and increased to (1.45 ± 0.295) × 10^3 MPa after 5 min drying at 40 ℃. E was data fitted by averaging ΔS/ΔD during the penetrating and pulling-out processes:

$$E = \frac{1}{2}\left(\frac{\Delta S_+}{\Delta D_+} + \frac{\Delta S_-}{\Delta D_-}\right) \qquad \text{(S6)}$$

The stress-to-displacement relationship represents a typical hysteresis loop (Figure S8), demonstrating a soft and viscoelastic material. Quick stimuli-responsiveness is fatigue-free and position-programmable, making the micro hydrogels suitable for the emerging micro-biomimetic or wearable devices.

Supplementary videos

Video S1. 532nm laser beam scanning in photoresist

Video S2. Swelled microlenses by dropping water

Video S3. Single swelled microlens

Video S4. pH responsiveness

Video S5. Shrinkage of hydrogel

Video S6. Leg moving of a spider-shaped hydrogel by laser focus

Video S7. No obvious motion when laser scans spider in air

Video S8. Tail swelling of a tadpole-shaped hydrogel

Video S9. Tail shrinkage of a tadpole-shaped hydrogel
